# Chrysanthemum *CmHSFA4* gene positively regulates salt stress tolerance in transgenic chrysanthemum

**DOI:** 10.1111/pbi.12871

**Published:** 2018-01-22

**Authors:** Fei Li, Huanru Zhang, Husheng Zhao, Tianwei Gao, Aiping Song, Jiafu Jiang, Fadi Chen, Sumei Chen

**Affiliations:** ^1^ College of Horticulture Nanjing Agricultural University Nanjing China

**Keywords:** salinity, CmHSFA4, chrysanthemum, overexpression, ionic, ROS

## Abstract

Salinity‐induced Na^+^ toxicity and oxidative stress hamper plant growth. Here, we showed that expression of the chrysanthemum *CmHSFA4*, a homologue of the heat‐shock factor *AtHSFA4a*, is inducible by salt and localizes to the nucleus. It is a transcription activator binding with HSE. Chrysanthemum overexpressing *CmHSFA4* displayed enhanced salinity tolerance by limiting Na^+^ accumulation while maintaining K^+^ concentration, which is consistent with the up‐regulation of ion transporters *CmSOS1* and *CmHKT2*. Additionally, the transgenic plants reduced H_2_O_2_ and O_2_^∙−^ accumulation under salinity, which could be due to up‐regulation of ROS scavenger activities such as SOD, APX and CAT as well as *CmHSP70*, *CmHSP90*. Together, these results suggest that *CmHSFA4* conferred salinity tolerance in chrysanthemum as a consequence of Na^+^/K^+^ ion and ROS homeostasis.

## Introduction

Soil salinization is one of the major issues threatening crop productivity worldwide as it affects plant growth by causing osmotic imbalance, mineral deficiency and overall toxicity (Parvaiz and Satyawati, 2014). To cope with salinity stress, plants tend to re‐establish ionic and ROS homeostasis. Regarding ionic homeostasis, it is important to maintain a low level of Na^+^ while maintaining a high concentration of K^+^ in the cytosol (Guan *et al*., 2013). The salt overly sensitive (SOS) signalling pathway is remarkable in the transport of toxic ions. *SOS1* encodes for a plasma membrane antiporter Na^+^/H^+^ which removes Na^+^ from cells (Rahman *et al*., 2016). HKT1, a member of the high‐affinity K^+^ transporters gene family, plays important roles as a Na^+^‐selective uniporter, under normal K^+^ concentration, HKT1 is mainly involved in Na^+^ unloading, and the major function of SOS1 is Na^+^ exclusion (Wang *et al*., 2014). They consequently play a crucial role in maintaining cellular ion homeostasis under salt stress by reducing the accumulation of Na^+^ and maintaining stable levels of K^+^ under salt stress (Ashraf and Sharif, 2008).

Except for ion toxicity, salt stress leads to the accumulation of high levels of reactive oxygen species (ROS). ROS, including superoxide (O_2_^∙−^), hydrogen peroxide (H_2_O_2_) and hydroxyl radicals (OH^∙−^), are continuously produced by aerobic metabolism in mitochondria, chloroplasts and peroxisomes in plants and can cause oxidative damage to proteins, DNA and lipids under stress (Akter, 2015). The balance of oxidative stress levels and ROS‐scavenging enzymes are directly related to ROS cellular toxicity (Mittler *et al*., 2011). Plant cells utilize antioxidant mechanisms to defend from the damage of ROS (Wrzaczek *et al*., 2013). Nonenzymatic ROS‐scavenging mechanisms include the major cellular redox buffers such as ascorbate, glutathione (GSH), ascorbic acid and carotenoids (Mittler, 2002). The enzymatic mechanisms mainly include superoxide dismutase (SOD), ascorbate peroxidase (APX) and catalase (CAT) (Sewelam *et al*., 2016). SOD converts hydrogen superoxide into hydrogen peroxide, which acts as the first line of defence to degrade the accumulating H_2_O_2_. APX and CAT convert hydrogen peroxide into water and subsequently detoxifies H_2_O_2_ by the ascorbate–glutathione cycle (Schmitt *et al*., 2014).

Heat‐shock factors (HSFs) are important regulators of cellular stress. HSF gene families are large, including 21 genes in Arabidopsis, 24 in tomato, 52 in soybean and more than 56 in wheat (Xue, 2013). HSFs can be subdivided into three classes, A, B or C, depending on their domains. They all share a conserved N‐terminal DNA‐binding domain, which is responsible for heat‐shock response element (HSE) recognition in the promoters of HSF target genes (Akerfelt *et al*., 2010). The hydrophobic heptad repeat region for oligomerization (HR‐A/B), which is located close to the DNA‐binding domain and mediates trimerization, is a prerequisite for their transcription factor activity (Lutz Nover *et al*., 2001). In response to biotic and abiotic stresses, HSF proteins have various roles as positive or negative regulators. Most of them are regulated by heat shock, especially *HSFA1*, *HSFA2* and *HSFA6* in tomato and wheat (Mishra *et al*., 2002). Furthermore, it was reported that *AtHSFA3* is regulated by *DREB2A* and enhances drought stress tolerance in Arabidopsis (Scharf *et al*., 2012); *FaHSFA2c* acted as a positive regulator conferring thermotolerance through the regulation of heat‐protective gene transcriptional expression in Arabidopsis and tall fescue (Wang *et al*., 2017). *LlHSFA1*, which interacts with *LlHSFA2*, enhanced thermotolerance in transgenic Arabidopsis overexpressing *LlHSFA1* (Gong *et al*., 2014); HSBs have no transcription activity and usually act as negative regulator. *AtHSFB1* repressed expression of *AtHSFA2*, *AtHSFA7a* and *AtHSFB2b* under moderate heat conditions (28 °C) in transgenic Arabidopsis (Ikeda and Ohme‐Takagi, 2011). *VpHSF1*'s overexpression lines reduced basal thermotolerance, increased acquired thermotolerance and reduced tolerance to osmotic stress in transgenic tobacco (Peng *et al*., 2012). However, HsfB1 represents a novel type of coactivator cooperating with class A HSFs (e.g. with tomato HsfA1; Bharti *et al*., 2004). Although HSFs have a wide array of members and complex responses to stress, the function of the HSFA4 group is not well known (Pérezsalamó *et al*., 2014). *TaHSFA4a* and *OsHSFA4a* enhanced Cd tolerance by up‐regulating metallothionein gene expression in rice plants (Shim *et al*., 2009); Co‐overexpression of *Helianthus annuus HaHSFA4a* and *HaHSFA9* enhanced the tolerance to dehydration and drastic oxidative stress, and the improved tolerance is accompanied with the accumulation of small heat shock proteins (sHSP) which are activated by *HaHSFA9* in transgenic tobacco (Personat, 2014). Arabidopsis *HSFA4A* was implicated in the regulation of responses to high light and oxidative stress by regulating the transcription of the *APX1* and *ZAT12* genes (Davletova *et al*., 2005). These data indicated that the HSFA4 group contributes to Cd stress, high light stress and oxidative stress. In addition to this, HSFA4 confers salt tolerance. Knockout plants of *Athsfa4a* are hypersensitive to salt stress because of the elevated hydrogen peroxide accumulation and lipid peroxidation under salinity (Pérezsalamó *et al*., 2014). HSFA4's function results in the enhanced expression of stress–response transcripts and regulates plant ROS homeostasis under stress.

Chrysanthemum, a major commercial ornamental plant, is readily subjected to salinity stress, which causes leaf chlorosis and causes serious damages to the plant's health. Improving salt tolerance of chrysanthemum will be critical to achieve a stable and sustainable production. Here, we isolated the *CmHSFA4* gene from Chrysanthemum. Chrysanthemum overexpressing *CmHSFA4* showed a regulation of salt tolerance by regulating ionic and ROS homeostasis. This study lays the foundations for chrysanthemum salinity improvements in the future.

## Results

### *CmHSFA4* sequence characteristics

**Figure 1:**
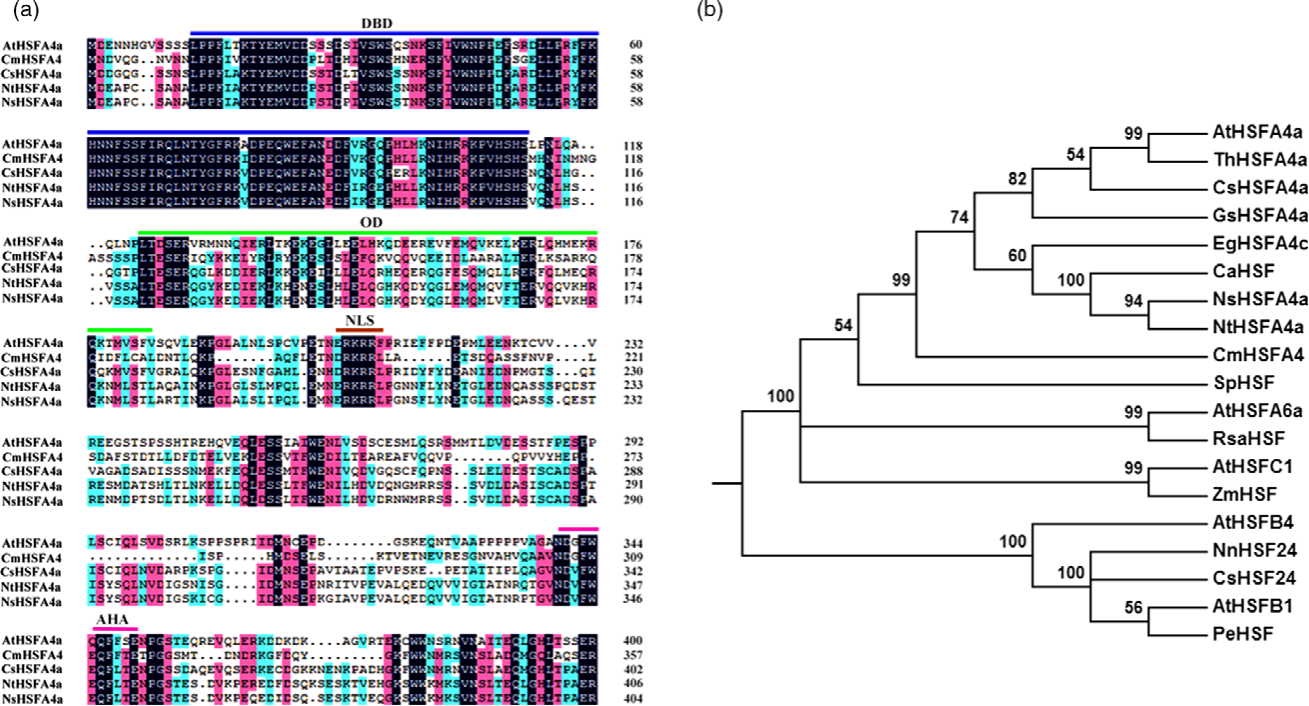
Deduced peptide sequences of CmHSFA4 and other HSF proteins. (a) Alignment of the putative amino acid sequence of CmHSFA4 with homologous proteins. Features of the sequence include a DBD domain, HR‐A/B region, NLS region and AHA motif, (b) Phylogenetic analysis of relationships between CmHSFA4 and HSF proteins from other plant species. The amino acid sequences were aligned with DNAMAN, and the phylogenetic tree was constructed using the neighbour‐joining method with MEGA 5.0. The sequence details are as follows: *AtHSFA4*(AT4G36990.1), *ThHSFA4a*(XP_010518819.1), *CsHSFA4a*(XP_006467595.1), *GsHSFA4a*(KHN06431.1), *EgHSFA4c*(XP;012849282.1), *CaHSFA4a*(XP_016557508.1), *NsHSFA4a*(XP_009785427.1), *NtHSFA4a*(XP_009590974.1), Sopim06(g072750.0.1), *AtHSFA6a*(AT5G43840), *RsaHSF* (1.0_00347.1_g00006.1), *AtHSFC1*(AT3G24520), *ZmHSF* (sc00747.1.g00320.1), *AtHSFB4*(AT1G46264), *NnHSF24*(010269874.1), *CsHSFA*‐4a(XP_006467595.1), *AtHSFB1*(AT4G36990), *PeHSF* (XP 011043547.1).

The *CmHSFA4* gene sequence consists of 1242‐bp with a 1074‐bp ORF encoding a 358 amino acid protein. It contains a conserved HSF‐DBD domain in its N‐terminal, a volatile AHA motif at the C‐terminal, and an intermediate HR‐A/B region and nuclear localization/export signal (NLS/NES) (Figure 1a). Phylogenetic analysis showed that CmHSFA4 showed a high similarly to other known HSFA4a, with an amino acid sequence similarity of 37.91% to *AtHSFA4a*, 43% to *NsHSFA4a* (XP_009785427.1) and 44% to *NtHSFA4a* (XP_009590974.1) (Figure 1b).

### CmHSFA4 localized to the nucleus

**Figure 2:**
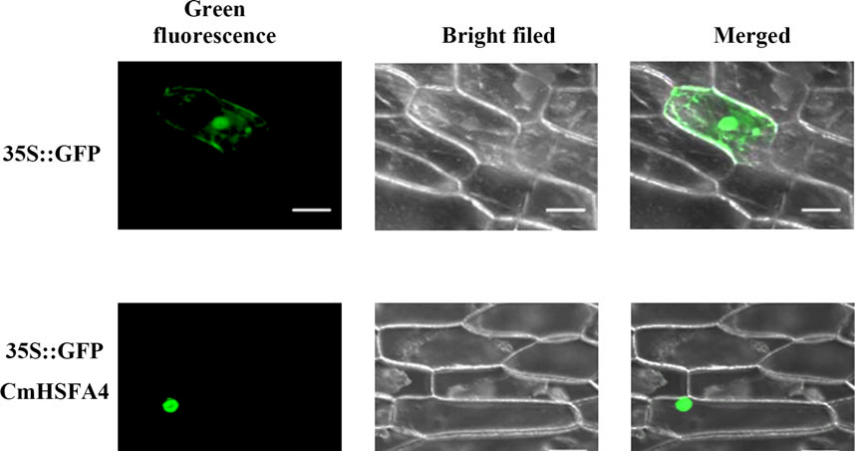
Subcellular localization of the CmHSFA4. Bar 100 μm.

To confirm the nuclear localization of CmHSFA4, CmHSFA4‐GFP fusion driven by the 35S promoter was introduced into onion epidermal cells. The GFP signal was mainly detected in the nucleus of cells transformed with pMDC43‐CmHSFA4‐GFP. For cells transformed with the positive control pMDC43‐GFP, GFP was expressed throughout the cells including the cytoplasm and nuclei (Figure 2).

### CmHSFA4 can activate transcription and bind with HSE

**Figure 3:**
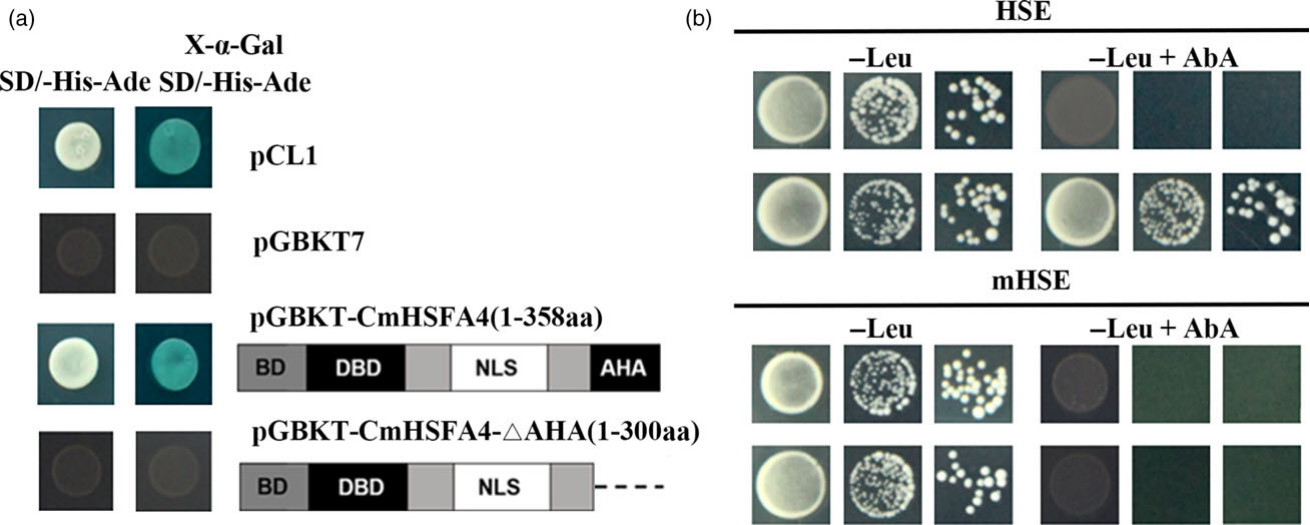
Transactivation analysis of CmHSFA4 and DNA‐binding assay. (a) The transcriptional activity analysis of CmHSFA4 in a yeast assay system, in which pCL1 is a positive control, pGBKT7 as a negative control, (b) assay of CmHSFA4 binding to HSE (CCAGAAGCTTCCAGAAGCC) or mHSE (CCAtAAGCTTaCA tAA GCC) using a yeast system. The lowest concentrations of Aureobasidin A (AbA) that limited the growth of yeast bait strains were 1 mg/mL.

Yeast containing the positive control pCL1‐pGBKT and those containing the pGBKT7‐CmHSFA4 construct grew well on SD/‐His‐Ade medium and became blue on SD//‐His‐Ade medium supplemented with x‐α‐gal, whereas the negative control pGBKT7 was unable to grow on the medium. Yeast cells harbouring pGBKT7‐CmHSFA4‐∆AHA, where the AHA motif was omitted, were unable to grow on the SD/‐His‐Ade medium (Figure 3a), suggesting that CmHSFA4 is a transcription activator and the AHA motif is important for its transcriptional activity. Cis‐element binding assays showed that CmHSFA4 could bind to HSE thereby activating HSE‐AUR1‐C to confer to Aureobasidin resistance, but the empty vector and the mutant HSE did not (Figure 3b).

### Inducible expression of *CmHSFA4* by salinity

**Figure 4:**
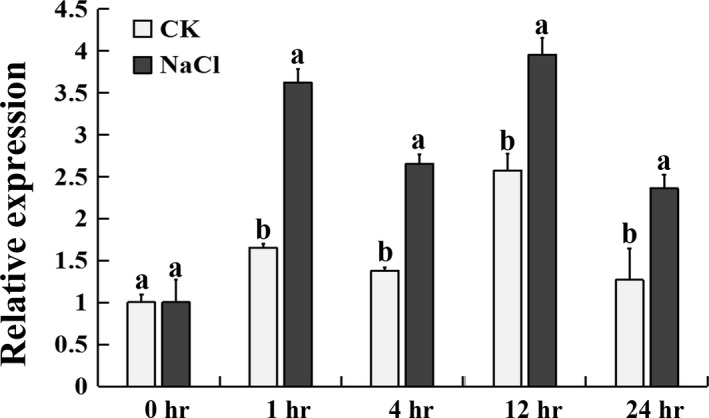
Expression of *CmHSFA4* in chrysanthemum plants under 200 mmol/L NaCl salinity treatment as revealed by quantitative real‐time PCR.

Under salinity stress conditions, *CmHSFA4*'s transcript expression level was induced to be 3.5‐folds of that of control at 1 h and remained significantly higher than that of control over 24 h (Figure 4), suggesting that *CmHSFA4* might be involved in the response of plant to salinity stress.

### *CmHSFA4* overexpression enhanced salinity tolerance of chrysanthemum

**Figure 5:**
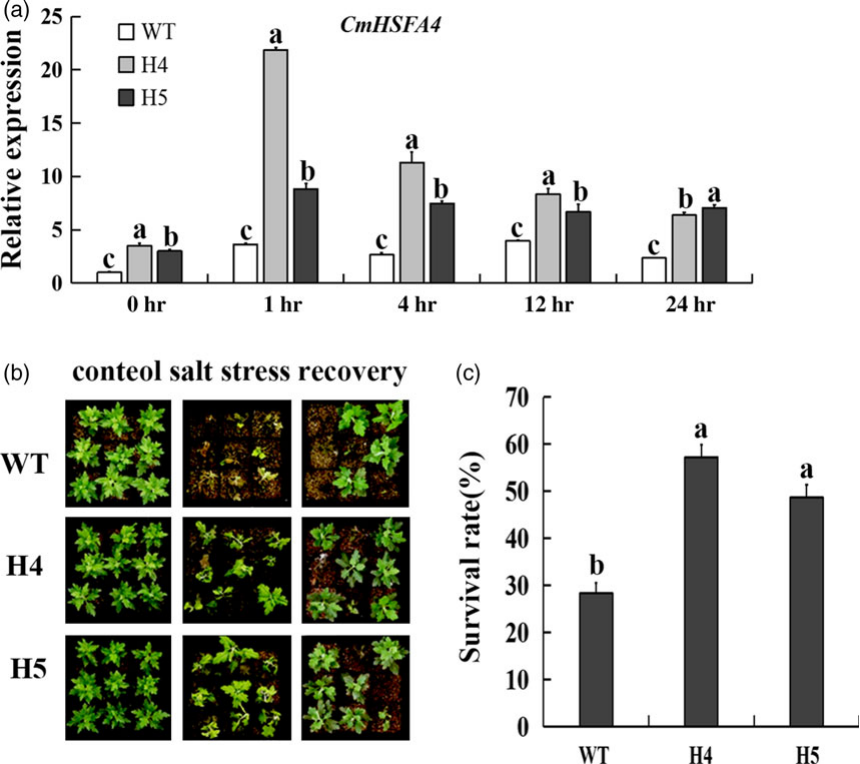
*CmHSFA4* overexpression enhanced salinity tolerance in chrysanthemum. (a) The expression level of *CmHSFA4* in WT and *CmHSFA4* OX lines H4, H5 plants under salinity treatment, (b) The phenotypic effect of watering with 200 mmol/L NaCl for 2‐weeks, followed by 2‐week recovery period, (c) Plant survival rate measured at the end of the recovery period. WT, wild‐type, H4 and H5, transgenic plants overexpressing CmHSFA4. Bars indicate standard error.

*CmHSFA4* transgenic chrysanthemum was successfully generated and validated by PCR (Figure S1a). The expression levels of *CmHSFA4* were higher in *CmHSFA4* OX (overexpressing) plants than that of WT (wild type) (Figure S1b). Two independent OX lines H4 and H5 exhibiting high transcript levels of *CmHSFA4* with a single copy of T‐DNA integration (Figure S1b,c) were selected for further salinity tolerance assay. The expression level of *CmHSFA4* in OX lines H4 and H5 was much higher than that in wild‐type plant under the salinity treatment (Figure 5a). Salinity tolerance of *CmHSFA4* overexpressing plants was assessed upon 200 mmol/L NaCl treatment for 7 days. *CmHSFA4* overexpressing plants H4 and H5 showed less stress damage compared with WT chrysanthemum plants. The top of *CmHSFA4* overexpressing plants remained green compared with WT plants and only the base leaves turned yellow after 7‐day salinity treatment, while the wild type became severely wilted, withered and some plants died after salinity treatment for 7 days (Figure 5b). The survival rate of H4 and H5 plants was 55.6% and 48.3%, while that of WT plants was only 28.0% (Figure 5c), indicating that overexpression of *CmHSFA4* enhanced salt tolerance of chrysanthemum.

### *CmHSFA4* overexpression retarded chlorophyll contents decrease under salinity stress

**Figure 6:**
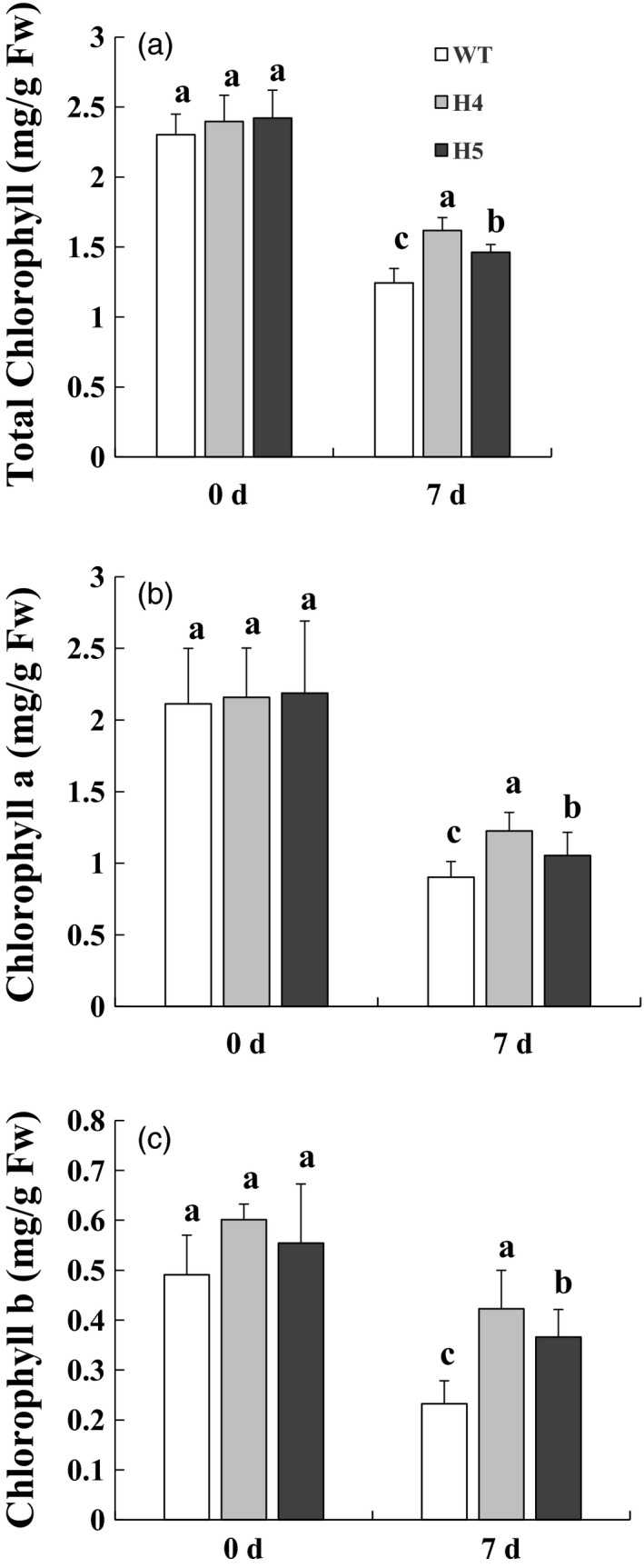
Estimation of chlorophyll contents in wild‐type and *CmHSFA4* overexpressing plants grown in the presence of 200 mmol/L NaCl. (a) Total chlorophyll, (b) chlorophyll a, (c) chlorophyll b contents of wild‐type, *CmHSFA4* transgenic H4 and H5 plants after salt treatment. Bars indicate the standard error.

Under normal growth conditions, the chlorophyll contents in OX lines H4 and H5 were almost comparable to those in the WT plants (Figure 6). Total chlorophyll contents in OX lines and WT plants both decreased upon 200 mmol/L NaCl treatment for 7 days. The total chlorophyll content reduced by 45.9% in WT plants but 32.4% and 39.6% in OX lines H4 and H5 on day 7 (Figure 6a). Consistently, chlorophyll a content in OX lines H4 and H5 showed 43.2% and 51.6% reduction, while 57.3% in wild‐type plants under salinity (Figure 6b). Similarly, chlorophyll b content in WT plants reduced by 52.7%, while 29.7% and 34.0% reduction in H4 and H5 plants (Figure 6c), indicating that overexpression of *CmHSFA4* protected chlorophyll from degradation by salinity stress.

### *CmHSFA4* overexpression balanced ion homeostasis and changed salinity stress‐related gene expression

**Figure 7:**
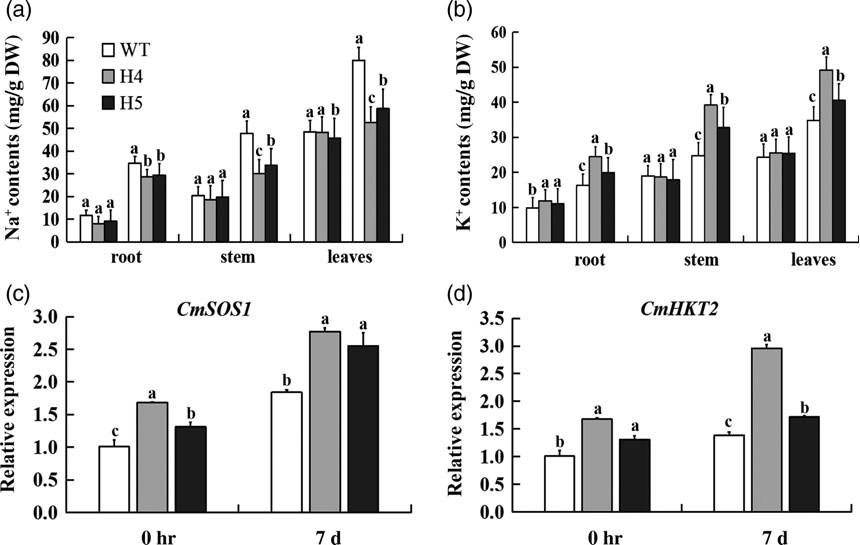
Na^+^ and K^+^ contents in wild type and transgenic overexpressing of *CmHSFA4* grown in the presence of 200 mmol/L NaCl. (a) Na^+^ content, (b) K^+^ content, in various parts of the plant. Columns marked with different lower case letters indicate a significant difference from the WT's performance, (c) *CmSOS1*, (d) *CmHKT2*, Relative expression levels in wild‐type and transgenic ‘Jinba’ after salt treatment (*P* < 0.05).

To assess the effect of *CmHSFA4*'s overexpression on ion homeostasis, six to eight leaf‐stage transgenic and wild‐type plants were subjected to 200 mmol/L NaCl for 7 days. Under nonstress growing conditions, there was little variation in the Na^+^ and K^+^ contents between the transgenic and wild‐type plants. On day 7, the Na^+^ contents of H4, H5 were significantly lower than those of WT plants, with 50.4% and 45.9% of WT plants in roots, 58.2% and 53.3% of WT plants in stems and 73.3% and 65.7% of WT plants in leaves (Figure 7a). Under salinity stress conditions, H4, H5 plants had a higher K^+^ content than that found in the WT plants. The K^+^ content in the roots of H4 was 48.3%, and H5 was 74.7% higher than the level in WT plants, the stem K^+^ contents, respectively, 59.2% and 72.2%, 55.2% and 74.2% in leaves (Figure 7b). At the same time, the transcript levels of ion homeostasis genes *CmSOS1* and *CmHKT2* in *CmHSFA4* were much higher than the WT with or without salinity (Figure 7c,d).

### *CmHSFA4* overexpression reduced ROS levels and activated ROS scavenger activities

**Figure 8:**
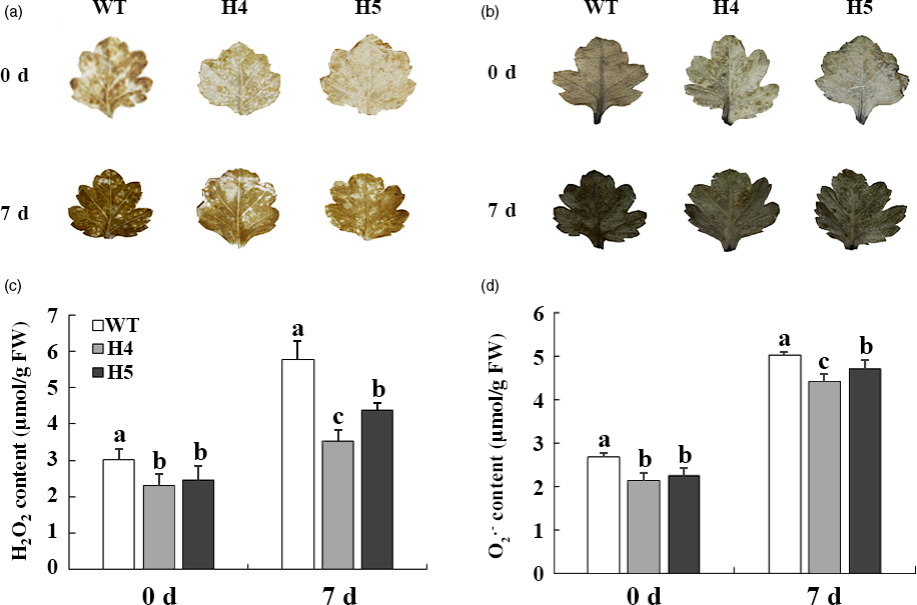
ROS contents of salinity‐stressed wild‐type ‘Jinba’ and *CmHSFA4* transgenic lines. (a) DAB staining (3,3′‐diaminobenzidine), (b) NBT staining, H_2_O_2_ and O_2_^∙−^ accumulation in control and salt‐treated chrysanthemum leaves visualized by DAB and NBT staining, which was used to monitor the ROS production in salt‐treated leaves, (c) leaf H_2_O_2_ content, (d) leaf O_2_^∙−^ content. Data represent means and standard errors of three replicates.

For *in vivo* localization and quantification of H_2_O_2_ and O_2_^∙−^, WT and transgenic leaves were stained with diaminobenzidine (DAB) (dark brown) and nitrotetrazolium blue chloride (NBT) (dark blue). *CmHSFA4* overexpressing exhibited clearly lower intensities of DAB and NBT staining in leaves compared to WT plants, reflecting a low level of H_2_O_2_ and O_2_^∙−^ accumulation (Figure 8a,b). In contrast, under normal growth conditions, H_2_O_2_ levels were comparable in WT and *CmHSFA4*ox plants, treatment with 200 mmol/L NaCl for 7 days increased the amount of H_2_O_2_ by 88% in the wild type but only by 59% and 63% in HSFA4ox plants. Consistently, O_2_^∙−^ contents in H4 and H5 plants increased by 80.2% and 92.7% but 107% in WT under salinity (Figure 8c,d).

**Figure 9:**
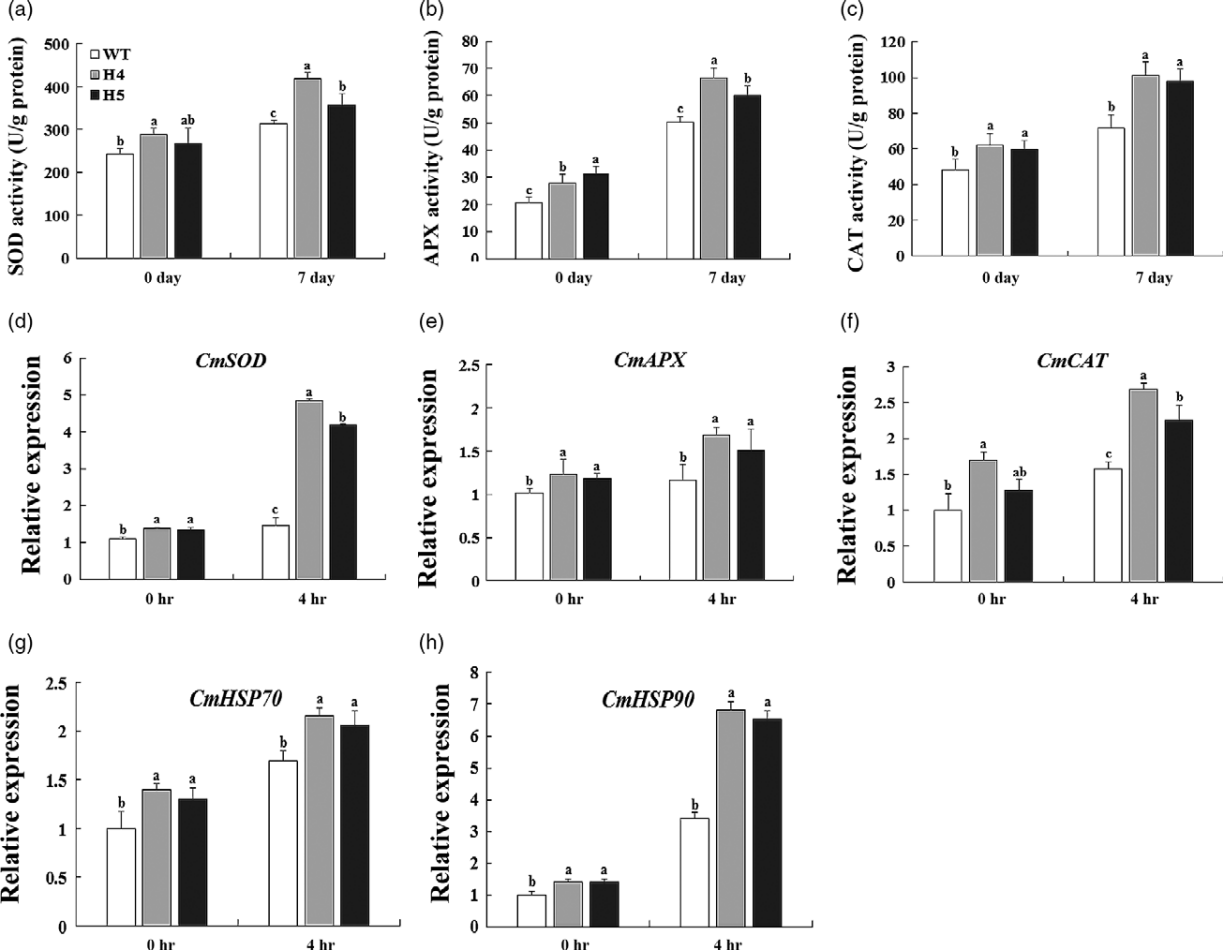
ROS scavenger activities and related gene expressions in wild‐type and *CmHSFA4* transgenic lines. (a) Leaf superoxide dismutase (SOD) activity, (b) leaf ascorbate peroxidase (APX) activity, (c) leaf catalase (CAT) activity, (d–h) expression of stress‐related genes in WT and the *CmHSFA4* transgenic lines (H4 and H5). Data represent means and standard errors of three replicates.

To further elucidate the role of *CmHSFA4* in ROS homeostasis, we examined the activities of ROS scavengers and found SOD, APX, CAT in OX plants were higher compared with WT at day 0 and day 7 of the salinity stress treatment (Figure 9a–c). The expression levels of ROS homeostasis‐associated genes including *CmSOD*, *CmAPX* and *CmCAT* (ROS scavenger encoding genes), *CmHSP70* and *CmHSP90* (heat‐shock protein genes) were examined by real‐time PCR analysis. Small differences between WT and OX plants under nonstress conditions were observed. Under salinity stress, ROS scavenger genes and *CmHSPs* were up‐regulated in both WT and OX plants, and the expression levels in OX plants were always higher than those in WT plants (Figure 9d–h).

## Discussion

### *CmHSFA4* confers salinity tolerance in chrysanthemum

Members of the HSF family are involved in various stresses such as heat stress, drought, anoxia, cold, high light and pathogen systemic acquired resistance (Guo *et al*., 2016). A few members of class A HSFs have been reported to play a role in salinity tolerance. For example, *AtHSFA2* increased salt/osmotic stress tolerance of Arabidopsis (Ogawa, 2007); *OsHSFA2e* enhanced tolerance to high‐salinity stress in transgenic Arabidopsis (Yokotani and Oda, 2008). In addition, *AtHSFA4a* conferred salt tolerance and oxidative stress (Pérezsalamó *et al*., 2014). In the present study, overexpressing the class A HSFA4 homolog *CmHSFA4*, probably a single copy gene in chrysanthemum (Figure S3), enhanced tolerance to salinity in chrysanthemum, indicating *CmHSFA4* is functionally conserved for salinity tolerance. The expression level of *CmHSFA4* in OX lines was much higher than that in WT plant under the salinity treatment (Figure 5a), which might be a consequence of the posttranscriptional stabilization of *CmHSFA4* transcripts by salinity stress. Similarly, posttranscriptional stability of *SOS1* in 35S:SOS1 overexpressing plants under salt stress has been previously described (Chung *et al*., 2008). We supposed that *CmHSFA4* overexpression contributed to the acquired tolerance of OX lines H4, H5 plants to salinity, while an induction of *CmHSFA4* by salinity in WT plants might confer to a basal tolerance of WT plants to salinity.

We also testified whether *CmHSFA4* contributed to osmotic adjustment; however, no significant differences in wilting, relative water contents and osmotic potential between WT and OX lines H4, H5 subjected to PEG6000 treatment have been observed (Figure S4), inferring that *CmHSFA4* might not contribute to osmotic adjustment.

### *CmHSFA4* enhanced tolerance to salinity in chrysanthemum is a consequence of ion homeostasis

Ion transport is the basic factor determining salinity tolerance. Along with ion uptake and transport, sequestration and extrusion, Na^+^‐K^+^ homeostasis governs the principal mechanisms of salt tolerance in plants (Vinod *et al*., 2013). Transcript accumulation of ion homeostasis‐associated genes served as the main regulators under salinity (Parvaiz and Satyawati, 2014). *SOS1*‐overexpressing transgenic tobacco and Arabidopsis accumulated less Na^+^ than WT plants under salt stress by limiting loading Na^+^ into the xylem and controlling long‐distance Na^+^ transport from xylem stream (Yue *et al*., 2012). *SOS1* retrieves Na^+^ from the xylem stream under severe salt stress (Shi *et al*., 2002). Under salinity, HKT1 primarily prevents Na^+^ overaccumulation in shoots via a downward stream of phloem in Arabidopsis (Horie *et al*., 2009). Under the low K^+^ with salt stress, *AtSOS1* functions in loading Na^+^ into the xylem to keep a relatively low level in surrounding parenchyma cells (Wang *et al*., 2014). *OsHKT2* acts as a Na^+^‐K^+^ symporter in tobacco cells and mediates a large Na^+^ influx into K^+^‐starved roots for growth (Jabnoune *et al*., 2009). In this present study, we found that *CmHSFA4* enhanced salt tolerance is possibly associated with the re‐establishment of ionic homeostasis. Compared to wild type, overexpressing *CmHSFA4* enhanced the ability of exporting Na^+^ under salinity conditions, which resulted in a lower accumulation in the roots, stems and leaves of OX plants. In contrast, K^+^ accumulation was distinctly enhanced in the roots, stems and leaves in the *CmHSFA4* overexpressing plants (Figure 7a,b). Consistently with the ion contents, *CmSOS1* and *CmHKT2* were up‐regulated in OX plants compared to the WT plants (Figure 7c,d). These data indicated that the overexpression of *CmHSFA4* worked to restrain the accumulation of Na^+^ and facilitate the absorption of K^+^ to detoxify the ionic toxicity caused by salinity. To our knowledge, *PeHSF* overexpressing tobacco enhanced salinity tolerance, while the re‐establishment of ionic homeostasis has not been affected in transgenic plants during the period of salt stress (Shen *et al*., 2013). This infers that members of HSF of different species may employ different strategies to cope with salinity stress. The mechanism through which HSFs regulate the Na^+^/K^+^ balance directly remains to be elucidated in further studies.

### *CmHSFA4* enhanced tolerance to salinity in chrysanthemum by regulating ROS homeostasis

ROS is another hampering factor for plant growth caused by high salinity. Salinity stress causes a significant accumulation of ROS in *sos1‐1* and *rcd1‐1* mutants leading to serious damage to Arabidopsis (Katiyaragarwal *et al*., 2006). Compared with wild type, *CmHSFA4* overexpressing plants suffered less ROS toxicity. Similarly, in *Athsfa4a* mutant plants, the H_2_O_2_ content was much higher than WT, which can be restored by *AtHSHA4a*, and the accumulation of H_2_O_2_ in *AtHSHA4a* overexpressing plants under salinity is less than that of WT (Guo *et al*., 2016). In response to stress‐triggered ROS production, it is important to keep a steady ROS level in the cells (Azarabadi *et al*., 2017). One strategy adopted by plants involves ROS scavengers such as SOD, APX and CAT to minimize ROS damage under salinity stress (Pan *et al*., 2006). Previous studies demonstrated the relationship between HSF and ROS‐scavenging enzymes. *ZmHsf06* enhances salt stress tolerance of transgenic Arabidopsis with higher SOD and POD activities (Li *et al*., 2015a). *AtHSFA2* regulated heat and oxidative stress with the induction of the *APX* expression (Li *et al*., 2005); *PeHSF* improved CAT activities under the salt stress (Shen *et al*., 2013). In the present study, the data showed that the activities of SOD, APX, CAT in OX plants were higher than those in WT under nonstressed or salt‐stressed condition (Figure 9a–c), indicating *CmHSFA4* conferred salinity tolerance is partially due to activating ROS scavengers.

Chloroplasts are particularly vulnerable to ROS induced damage (Gupta and Berkowitz, 1987). Salt stress affects mRNA editing in chloroplasts (Rodrigues *et al*., 2017). This is consistent with the observation in Salicornia brachiata stress‐related protein (*SbSRP*) transgenic tobacco with enhanced tolerance to salt stress (Udawat *et al*., 2017). Leaves of the salinity‐stressed wild‐type chrysanthemum plants were more affected by chlorosis and contained less chlorophyll than the *CcSOS1* transgenic plants (Gao *et al*., 2016). Similarly, in present study, Chl a, Chl b and total chlorophyll contents were decreased under saline conditions, while *CmHSFA4* overexpressing plants maintained higher contents of chlorophyll compared to the WT plants under saline stress (Figure 6), we supposed that *CmHSFA4* overexpression might prevent chlorophyll from the ROS damage to some extent.

A relationship between ROS and HSP has been identified (Timperio *et al*., 2008). Transcriptional reprogramming, the binding of HSF to HSE in the promoters of *HSP* genes, is essential for the induction of expression of plant *HSP* genes (Hua, 2009). *AtHSFA4a* enhanced salinity tolerance of Arabidopsis through regulating the MPK3 and MPK6 mitogen‐activated protein kinase pathway, led to the transcriptional activation of the *HSP 17.6A* gene. Class A HSF harboured a conversed DBD domain which specifically binds to heat stress elements (HSEs: (5′‐AGAAnnTTCT‐3′)) (Guo *et al*., 2016). Here, CmHSFA4 also has a conserved DBD domain, and the Y1H assay showed that it could bind to the HSE element (Figure 3b). In addition, CmHSFA4 has transcription activity in yeast cells depending on the AHA motif (Figure 3a), which is similar with that of AtHSFA4a, suggesting CmHSFA4 could activate the expression of those genes whose promoter regions harbour the HSE element. Transcriptional induction of *CmHSP70* and *CmHSP90* in OX plants was observed in nonstressed and stressed conditions, suggesting these two genes might be the direct target gene of CmHSFA4; however, *in vivo* evidence should be provided before we can make a conclusion. Despite this hypothesis, an elevation of *CmHSP70* and *CmHSP90* in OX plants should also contribute to limiting ROS damage.

In addition, *SOS1* not only takes part in ion homeostasis but also reduces ROS level under salinity stress. The durum wheat *TdSOS1* improves oxidative stress tolerance of overexpressing Arabidopsis plants (Feki *et al*., 2017). Cross‐talk between ion homeostasis and oxidative stress pathways has been previously described, where *AtSOS1* up‐regulated the oxidative stress tolerance gene *Fe‐SOD* expression through oxidative tolerance gene *RCD1* (Katiyaragarwal *et al*., 2006). We have previously shown that *CmSOS1*, *CcSOS1* conferred salinity tolerance in chrysanthemum via balancing the Na^+^/K^+^ ratio and maintaining stable ROS levels (An *et al*., 2014; Gao *et al*., 2016; Li *et al*., 2015b). Here, an elevation in *CmSOS1* and *CmSOD* was observed in OX plants, suggesting that *CmHSFA4* might play an intermediate role in the cross‐talk between ion homeostasis and oxidative stress.

## Experimental procedures

### Plant materials and grow conditions

The chrysanthemum cultivar ‘Jinba’ was obtained from the Chrysanthemum Germplasm Resource Conservation Centre, Nanjing Agricultural University, China. Seedlings of similar size at six to eight leaf stage were planted in pots using a 1 : 3 (v/v) mixture of soil and vermiculite and cultivated in a greenhouse under day and night temperatures of 25/18 °C, respectively, and a 14‐h light/10‐h dark photoperiod with a relative humidity of 70%.

### Isolation and sequence analysis of *CmHSFA4* cDNA

Full‐length cDNA was isolated with the previously reported gene‐specific primers CmHSFA4‐F/R (Table S1; Xia *et al*., 2014). The CmHSFA4 amino acid sequence was aligned with its homologs using the DNAMAN 5.2.2 software and BLAST software online (http://www.ncbi.nlm.gov/blast). A phylogenetic tree was constructed using the neighbour‐joining method with MEGA 5.2.2.

### Subcellular localization of CmHSFA4

To detect the subcellular localization of CmHSFA4, we generated the p35S::GFP‐CmHSFA43 fusion construct. The CmHSFA4 ORF was amplified by PCR using the primer set CmHSFA4‐1A‐F/R (Table S1) harbouring the *Xho* I and *Not* I sites. Both the amplified fragment and pENTR™1A were digested with *Xho* I and *Not* I; then the corresponding bands were recovered and ligated into pENTR™1A to yield the expression vector pENTR™1A‐CmHSFA4. Then, CmHSFA4 was introduced to the vector pMDC43 (Invitrogen) by LR reaction between pMDC43 and pENTR™1A‐CmHSFA4. The plasmids including pMDC43‐CmHSFA4 and empty pMDC43 vector (negative control) were introduced into onion epidermal cells by a helium‐driven particle accelerator (PDS‐1000; Bio‐Rad, Hercules, California, USA). Onion epidermal cells were incubated for 16 h at 22 °C in the dark before observation of GFP signal via confocal laser scanning microscopy.

### Transcriptional activity analysis and DNA‐binding assay of CmHSFA4

For transcriptional activity analysis, CmHSFA4 was cloned into the yeast expression vector pGBKT7 by LR reaction using pENTR™1A‐CmHSFA4 constructs to generate pGBKT7‐CmHSFA4. The fragment CmHSFA4‐∆AHA, which lacked AHA motif, was amplified using the Phusion High Fidelity PCR Kit (New England Biolabs, Ipswich, Massachusetts, USA) with the prime pair CmHSFA4‐AHA‐F/R (Table S1). Both the PCR products and pGBKT7 vector were digested with *Bam*H I and *Xoh* I, and gel recovered and ligated to yield pGBKT7‐CmHSFA4‐∆AHA. The pGBKT7‐CmHSFA4 construct, pGBKT7‐CmHSFA4‐∆AHA, pCL1 (positive control) and pGBKT7 (negative control) were introduced into Y2HGold yeast cells. Transformants carrying either pGBKT7‐CmHSFA4, pGBKT7‐CmHSFA4‐∆AHA or pGBKT7 were selected on SD/‐Trp medium, whereas pCL1 was selected on SD/‐Leu medium.

To examine the DNA‐binding ability of CmHSFA4, CmHSFA4 was cloned into the yeast expression vector pGADT7. Y1H assay was performed to examine the DNA‐binding ability of CmHSFA4 to HSE using the Matchmaker Gold Yeast One‐Hybrid Library Screening System (Clontech, Mountain View, California, USA) as described in the manufacturer's instructions. A HSE consensus sequence element or its mutant form was synthesized with restriction enzyme digestion and cloned into the pAbai vector carrying the AUR1‐C gene (Song *et al*., 2015), kindly provided by Dr. Daolong Dou (Nanjing Agricultural University). Bait yeast cells were then transformed with the pGADT7‐CmHSFA4 while the empty vector pGADT7 was used as a negative control.

### Expression profile of *CmHSFA4* under salinity stress

For salinity treatment, plants at the six to eight leaf stage were subjected to 200 mmol/L NaCl, the 3rd leaf (counted from the apex) was harvested at 0, 1, 4, 12 and 24 h after salinity treatment for expression profile analysis of *CmHSFA4*. Three biological replicates of each experiment were conducted.

### Generation of *CmHSFA4* overexpressing chrysanthemum

The 35S::CmHSFA4 plasmid was transformed into the *Agrobacterium tumefaciens* EHA105 strain using the freeze–thaw transformation method. The transformation of chrysanthemum was performed as previously described (Li *et al*., 2015b). After regeneration, DNA was extracted from putative transgenic chrysanthemum plants and wild‐type (WT) plants using the Multisource Genomic DNA Miniprep kit (Axygen). The regenerating resistant plants were obtained using PCR with the primer pair Hyg‐F/R (Table S1). The RNA of the putative transgenic and control plants was extracted using a Quick RNA isolation Kit (Waryong) and reverse‐transcribed with the reverse transcription M‐MLV (TaKaRa, Tokyo, Japan). The qRT–PCR using SYBR Premix Ex Taq TM II (Tli RNaseH Plus) was employed to analyse the expression of *CmHSFA4* with the primer pair CmHSFA4‐RT‐F/R (Table S1). The primer pair CmEF1ɑ‐F/R (Table S1) was used to amplify the reference gene *CmEF1*α (KF305681). Transcription data with three biological replicates were calculated using the 2^−∆∆Ct^ method, and the expression level of WT was set as the basal.

DNA‐positive lines H4 and H5 with highest expression levels of *CmHSFA4* were subjected to DNA gel blotting. The details of DNA gel blotting are included in the Supplemental materials and Figure S2. The *CmHSFA4* transcript levels in transgenic plants over 24 h under 200 mmol/L NaCl salinity stress were quantified using procedures mentioned above.

### Salinity tolerance of *CmHSFA4* overexpressing chrysanthemum

For the salinity tolerance assay, the *CmHSFA4* overexpressing plants H4, H5 and wild‐type chrysanthemum at six to eight leaf stage were irrigated with 200 mmol/L NaCl for 2 weeks. After treatment, plants were removed from the soil, washed with distilled water, replanted in a fresh mixture of soil and vermiculite (1 : 1, v/v) and left to recover for 2 weeks (Li *et al*., 2015b). The survival rate of the transgenic and the WT plants was calculated. The experiment included three biological replicates, each replicate with 15 seedlings.

### Chlorophyll quantification in *CmHSFA4* overexpressing chrysanthemum under salinity

Chlorophyll contents of leaves from entire plant of WT and OX lines H4, H5 plants were determined at day 0 (before salinity treatment) and day 7 after salinity treatment as described by Arnon (1949) with minor modifications. Briefly, approximately 0.1 g (fresh weight) of leaves was incubated in 5 mL ethanol and acetone mixture (1 : 2, v/v) for 48 h in the dark, and then, the absorbance of the supernatant was analysed using a DU 800 UV/Vis spectrophotometer (Beckman Coulter, California, CA), scanning at 665, 649 nm, respectively. The experiment was repeated three times. Each replicate contained five seedlings.

### Na^+^ and K^+^ contents in *CmHSFA4* overexpressing chrysanthemum

To estimate Na^+^ and K^+^ content, plants were subjected to 200 mmol/L NaCl treatment for 7 days (An *et al*., 2014). Roots, stems and leaves were harvested separately on day 7, baked at 80 °C for 3 days, and 0.1 g dry sample was digested in 2 mL 10 mmol/L HNO_3_ and then metered volume to 10 mL by distilled water. Na^+^ and K^+^ contents were measured using an Optima 2100DV inductively coupled plasma optical emission spectrometer (Gao *et al*., 2004). The experiment was repeated three times.

### ROS production in *CmHSFA4* overexpressing chrysanthemum

Physiological traits of WT and H4, H5 plants were measured at day 0 (before salinity treatment) and day 7 of the salinity test. The quantification of H_2_O_2_ and O_2_^∙−^ levels was determined following the previously described method. Briefly, approximately 0.5 g of leaf tissues was homogenized with 5 mL 0.1% (w/v) TCA (trichloroacetic acid) in ice bath. The homogenate was centrifuged at 12 000 × ***g*** for 15 min, and 0.5 mL of the supernatant was added to 0.5 mL 10 mmol/L potassium phosphate buffer (pH 7.0) and 1 mL 1 mmol/L KI. The absorbency of supernatant was read at 390 nm. The content of H_2_O_2_ was given on a standard curve. Contents of O_2_^∙−^ were measured by hydroxylamine reaction. Approximately 1.0 g of leaf tissues was homogenized with 250 mmol/L phosphate buffer (pH = 8) containing with 10 μmol/L PLP (pyridoxal 5‐phosphate monohydrate), 1 mmol/L Na_2_EDTA and 5 mmol/L DTT in ice bath. The homogenate was centrifuged at 10 000 × ***g***, 4 °C for 25 min. The absorbency of supernatant was read at 530 nm. The content of O_2_^∙−^ was given on a standard curve (Velikova *et al*., 2000; Wang *et al*., 1990). Diaminobenzidine (DAB) and nitrotetrazolium blue chloride (NBT) stainings were used to detect the accumulation of H_2_O_2_ and O_2_^∙−^ in the transgenic chrysanthemum plants as previously described (Korasick *et al*., 2010). After overnight treatment with DAB and NBT separately, the stained leaves were cleared by boiling in 80% ethanol and then destained overnight in absolute ethanol. Representative phenotypes were photographed, and the experiment included three repeats using three different plants for each repeat.

### ROS scavenger enzymes activities in *CmHSFA4* overexpressing chrysanthemum

The activities of SOD, APX and CAT were assessed as previously described (Aebi, 1983; Fatima *et al*., 2011; Pan *et al*., 2006). SOD's activity of one‐unit was defined as the amount of enzyme required to cause a 50% inhibition of NBT. APX's activity was assayed from the decrease in absorbance at 290 nm as ascorbate was oxidized. The activity of CAT was determined as the reduction in enzymatic amount in 1 min. Enzymatic activities were expressed as enzyme units per g of protein. Each assay included three replicates of three different plants per line per time point.

### Quantification of salinity stress‐related genes in *CmHSFA4*‐transformed chrysanthemum

To analyse the expression levels of genes responsive to salinity stress, the third leaf from the apex of seedlings was collected. For expression profiles of *CmSOS1* and *CmHKT2*, leaves were sampled at 0 and 7 days after 200 mmol/L NaCl exposure. For ROS scavenger genes *CmSOD*, *CmAPX*, *CmCAT*, and *CmHSP70* and *CmHSP90* analysis, leaves were collected at 0 and 4 h after 200 mmol/L NaCl exposure. Each experiment included three biological replicates; samples collected from three individual plants at defined time points were pooled for RNA extraction. *CmEF1*ɑ was used as the reference gene. The sequences of all relevant primers are listed in Table S1.

### Statistical analysis

A one‐way analysis of variance, using Tukey's multiple range test (*P* = 0.05), was employed to identify treatment means that differed statistically. The SPSS v17.0 software (SPSS Inc, Chicago, IL) was used for all statistical analyses.

## Conflict of interest

The authors have no conflicts of interest to declare.

## Supporting information


**Figure S1** Identification of *CmHSFA4* overexpressing chrysanthemum.

**Figure S2** Diagram of the pMDC43‐CmHSFA4 construct, the structure of the *CmHSFA4* and restriction sites of digestion enzymes.

**Figure S3** DNA gel blotting analysis of genomic DNA isolated from wild type plants using a digoxigenin‐labeled *CmHSFA4* probe.

**Figure S4** Osmotic adjustment of WT and CmHSFA4 overexpressing chrysanthemum subjected to PEG6000 (20%) treatment.

**Table S1** Primer names and sequences used in this study.

Supplementary Legends
