# Supplementary figures and images for: Chrysanthemum CmHSFA4 gene positively regulates salt stress tolerance in transgenic chrysanthemum
Source: Plant Biotechnol J. 2018 Jan 22;16(7):1311–21. doi: 10.1111/pbi.12871 (PMC5999316; doi:10.1111/pbi.12871)

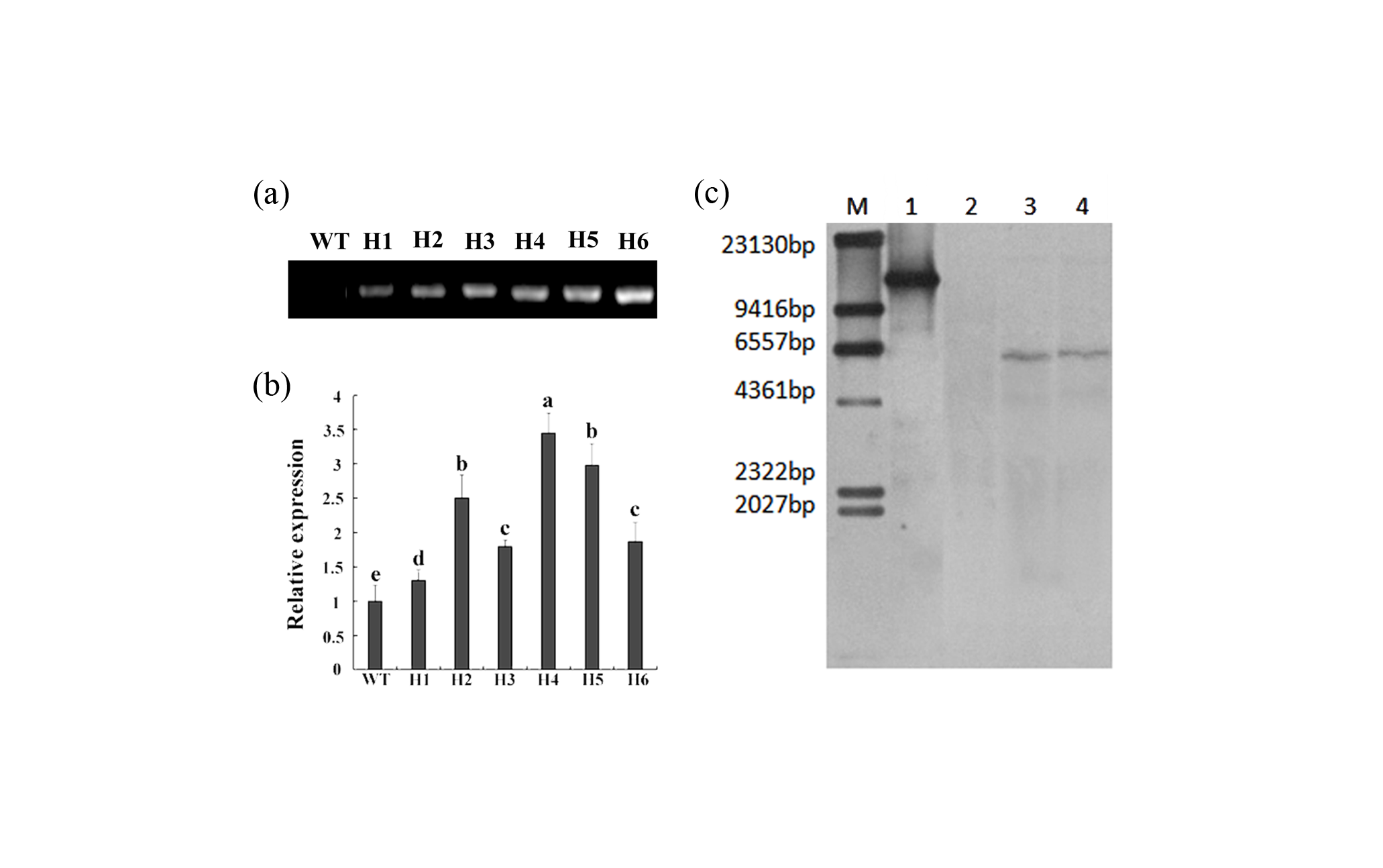

Supplement: Supplementary file 1 — Figure S1 Identification of CmHSFA4 overexpressing chrysanthemum. [file PBI-16-1311-s005.tif]

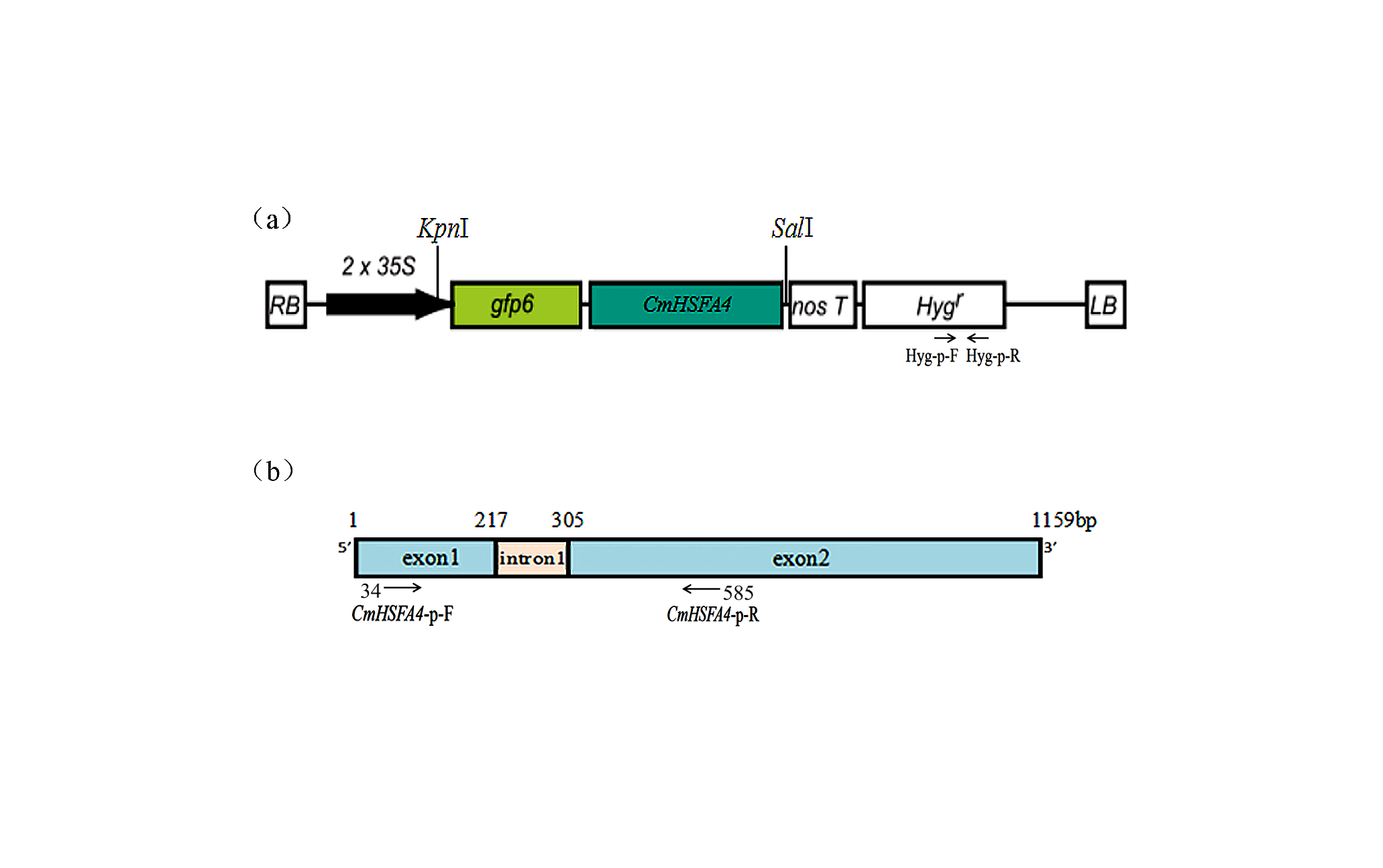

Supplement: Supplementary file 2 — Figure S2 Diagram of the pMDC43‐CmHSFA4 construct, the structure of the CmHSFA4 and restriction sites of digestion enzymes. [file PBI-16-1311-s004.tif]

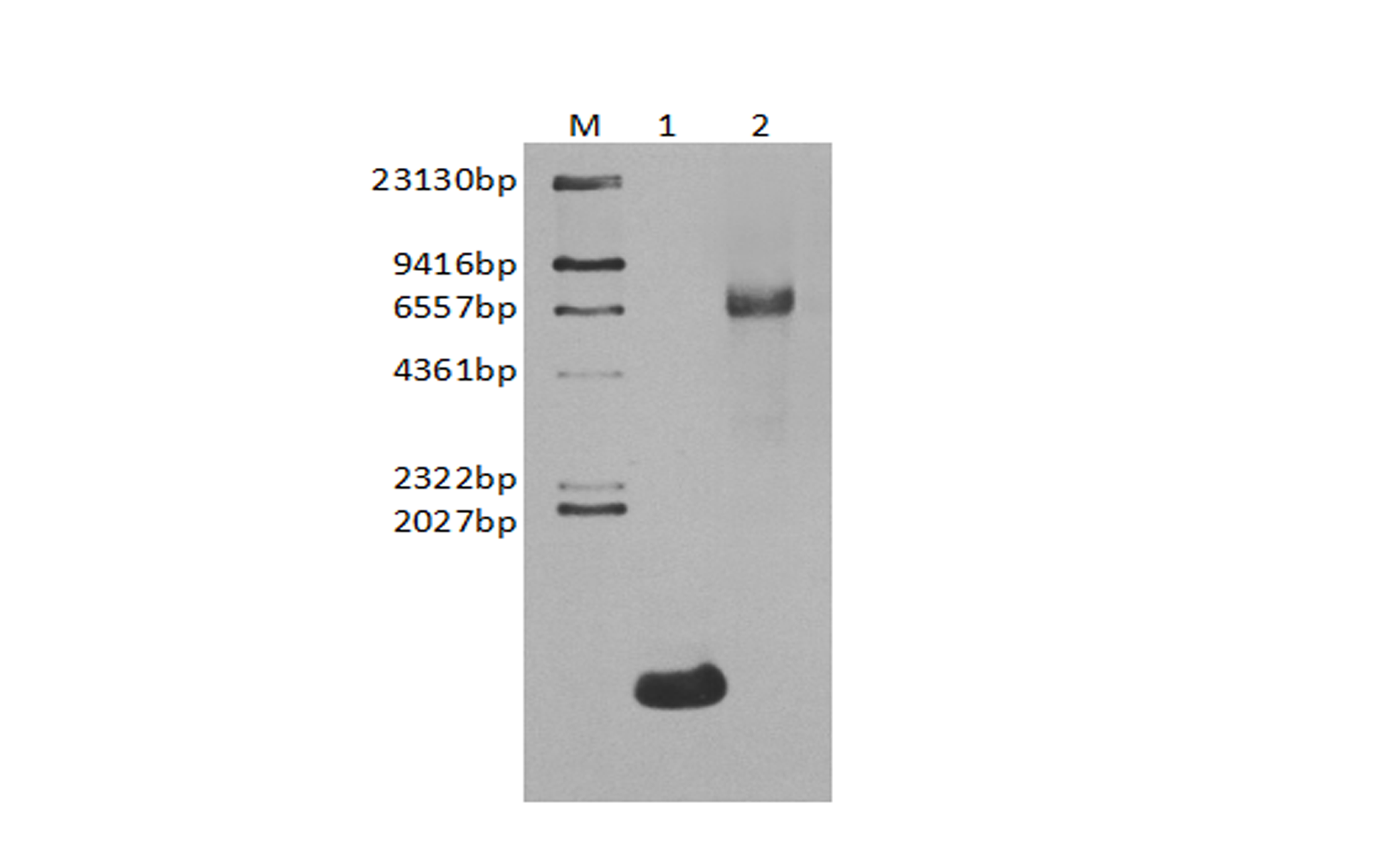

Supplement: Supplementary file 3 — Figure S3 DNA gel blotting analysis of genomic DNA isolated from wild type plants using a digoxigenin‐labeled CmHSFA4 probe. [file PBI-16-1311-s006.tif]

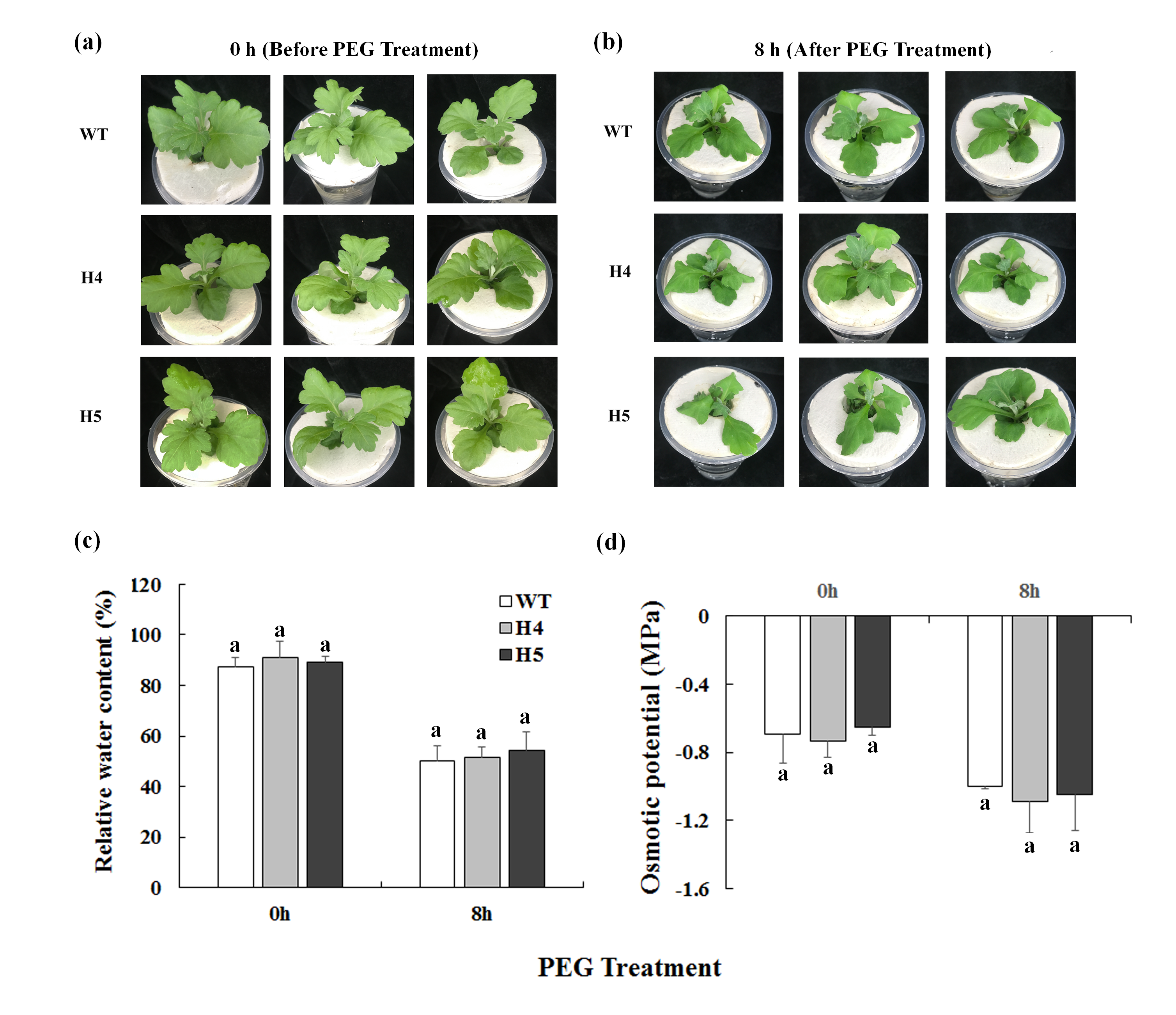

Supplement: Supplementary file 4 — Figure S4 Osmotic adjustment of WT and CmHSFA4 overexpressing chrysanthemum subjected to PEG6000 (20%) treatment. [file PBI-16-1311-s001.tif]
