# Supplementary material for: Chrysanthemum CmHSFA4 gene positively regulates salt stress tolerance in transgenic chrysanthemum
Source: Plant Biotechnol J. 2018 Jan 22;16(7):1311–21. doi: 10.1111/pbi.12871 (PMC5999316; doi:10.1111/pbi.12871)
Supplement: Supplementary file 5 — Table S1 Primer names and sequences used in this study. [file PBI-16-1311-s002.doc]

**Table S1 Primer names and sequences used in this study**

| Prime name | Sequence （5′ to 3′） |
| --- | --- |
| CmHSFA4-F | ATGAATGATGTTCAAGGTAATGTG |
| CmHSFA4-R | AGCTTGCTCAATCAGAAAGAACT |
| CmHSFA4-1A-F | CGGGGTACCGAATGAATGATGTTCAAGGTAA |
| CmHSFA4-1A-R | ATTTGCGGCCGCGAAGTTCTTTCTGATTGAGCAA |
| CmEF1α-F | TTTTGGTATCTGGTCCTGGAG |
| CmEF1α-R | CCATTCAAGCGACAGACTCA |
| Hyg-F | CTTCTACACAGCCATCGGTCCAG |
| Hyg-R | CGGAAGTGCTTGACATTGGGGAG |
| CmHSFA4-AHA-F | CTGCATATGCAAGCGGGTGTTAATGACGG |
| CmHSFA4-AHA-R | CGCGGATCCAGTTCTTTCTGATTGAGCAAGCTG |
| CmHSFA4-RT-F | AGTACGAGAAAGTGGGAATG |
| CmHSFA4-RT-R | TTTGATCTGCAAGACTATTAACACT |
| CmHKT2-F | GCGAGTTATGGGTTTG |
| CmHKT2-R | ACGAGGGGTAAAGAAT |
| CmSOS1-F | TGGGAGCCCTTCCATCAG |
| CmSOS1-R | TGCTTGCCCACTTCACCA |
| CmRBOHD-F | GATTAGGCAAGTTTCGGAGGAGTTA |
| CmRBOHD-R | CCCGGACTTAGTCCGGTCATA |
| CmRBOHF-F | GCTAAGGCGAGGCACA |
| CmRBOHF-R | TCGGAACGCCCTTATT |
| CmSOD-F | GCATGTCAACTGGTCCTCATTACAA |
| CmSOD-R | TCACGAAAGTAAGCCTTGCTATCCC |
| CmAPX-F | GACTAGGGAGCCGCTGAA |
| CmAPX-R | GGCGGAATGCAGGATC |
| CmCAT-F | TTGTTGGAGGACGAAG |
| CmCAT-R | CAGCGGCAAGATGTCC |
| CmHSP70-F | AAAGAACCAGAACCCGTGAC |
| CmHSP70-R | ATGTCAAAGCAGACTGTGATCTGTG |
| CmHSP90-F | GAACTTGGTGACACGGATGA |
| CmHSFA4-probe-F | CCTCCGTTTATCGTCAAG |
| CmHSFA4-probe-R | CGTGCTGCTAAGTCAATCT |
| Hyg-probe-F | GCTCCATACAAGCCAACCACG |
| Hyg-probe-R | CCTGCCTGAAACCGAACTGC |
