# Supplementary material for: Chrysanthemum CmHSFA4 gene positively regulates salt stress tolerance in transgenic chrysanthemum
Source: Plant Biotechnol J. 2018 Jan 22;16(7):1311–21. doi: 10.1111/pbi.12871 (PMC5999316; doi:10.1111/pbi.12871)
Supplement: Supplementary file 6 — Supplementary Legends [file PBI-16-1311-s003.docx]

**Figure S1.** Identification of *CmHSFA4* overexpressing chrysanthemum.

a. PCR detection of hpt in transgenic chrysanthemum. WT, Wild-type; H1, H2, H3, H4, H5 and H6, overexpressing transgenic plants of *CmHSFA4,* b. Relative expression levels of the CmHSFA4 gene in transgenic plants, c. Southern blot analysis of T-DNA integration using a digoxigenin-labeled *hpt* probe. M: DNA Molecular-Weight Marker, Lane1, Positive control (pMDC43-*CmHSFA4* construct digested with *Sal* I), Lane2, Wild-type plant digested with *Kpn* I (negative control), Lane3, *CmHSFA4* transgenic plants H4 digested with *Kpn* I, Lane4, *CmHSFA4* transgenic plants H5 digested with *Kpn* I.

**Figure S2.** Diagram of the pMDC43-CmHSFA4 construct, the structure of the *CmHSFA4* and restriction sites of digestion enzymes.

a. Diagram of the pMDC43-CmHSFA4 construct and recognition sites of *Kpn* I and *Sal* I, b. the structure of the *CmHSFA4*

**Figure S3.** DNA gel blotting analysis of genomic DNA isolated from wild type plants using a digoxigenin-labeled *CmHSFA4* probe.

M, DNA Molecular-Weight Marker, 1. Positive control (552 bp fragment of *CmHSFA4* gene), 2. DNA from wild type plants digested with *Kpn* I

**Figure S4.** Osmotic adjustment of WT and CmHSFA4 overexpressing chrysanthemum subjected to PEG6000(20%) treatment.

Morphology of plants before PEG6000 treatment (a) and 8h after PEG6000 treatment (b), relative water content (c) and osmotic potential (d) in WT and *CmHSFA4* overexpressing chrysanthemum under salinity treatment

**Table S1.** Primer names and sequences used in this study.
